# Impact of Nutritional Tea Polyphenols on Growth, Feed Efficiency, Biochemical Traits, Antioxidant Capacity, Haematological Parameters and Immunity in Coho Salmon (*Oncorhynchus kisutch*)

**DOI:** 10.3390/ani14142104

**Published:** 2024-07-18

**Authors:** Hairui Yu, Govindharajan Sattanathan, Leyong Yu, Lingyao Li, Yufang Xiao

**Affiliations:** 1 Key Laboratory of Biochemistry and Molecular Biology, Weifang Key Laboratory of Coho Salmon Culturing Facility Engineering, Institute of Modern Facility Fisheries, College of Biology and Oceanography, Weifang University, Weifang 261061, China; sattanathanphd@gmail.com (G.S.); leyong618@gmail.com (L.Y.); 2 Shandong Collaborative Innovation Center of Coho Salmon Health Culture Engineering Technology, Shandong Conqueren Marine Technology Co., Ltd., Weifang 261108, China; 980714742@163.com; 3 Conqueren Leading Fresh Science and Technology Inc., Ltd., Weifang 261205, China

**Keywords:** tea polyphenols, growth performance, antioxidant enzymes, *Oncorhynchus kisutch*

## Abstract

**Simple Summary:**

Phyto-based foods are becoming more and more popular worldwide, and people are consuming phyto-active components for purposes other than traditional health and nutrition. The study aimed to ascertain the impact of tea polyphenols (TPs) on fish. The outcomes showed that cold-water coho salmon exhibit improved growth, feed intake, and immunity. These results are helpful in promoting the development of novel feed additives for the aquaculture industry.

**Abstract:**

To evaluate the impact of nutritional tea polyphenols (TPs) on body composition, growth, biochemical markers, antioxidant capacity, and hemato-immunological levels, a ten-week feeding experiment was carried out on coho salmon (*Oncorhynchus kisutch*, 180.51 ± 0.15 g). The control group was fed a basal diet; the T1, T2, T3, and T4 groups were fed 0.005%, 0.01%, 0.02%, and 0.04% TPs, respectively. These results demonstrate that adding TPs significantly (*p* < 0.05) increased the coho salmon fish’s weight gain (WG), relative growth rate (RGR), condition factor (CF), feed efficacy (FE), daily growth rate (DGR), and specific growth rate (SGR). There was no discernible difference in the body compositions of the treated TPs and the control group (*p* > 0.05). In addition, the T3 group showed a significant (*p* < 0.05) decrease in GPT, LDL, HDL, TC, and CAT. Fish given a 0.02% diet containing TPs had significantly lower levels of malondialdehyde (MDA) in their liver; yet, the TP-treated groups had higher levels of SOD and CAT than the control (*p* < 0.05). The data analysis shows a significant rise in lysozyme, respiratory burst activity, bactericidal activity, and blood hematological parameters in the 0.01–0.04% TP groups. According to these findings, TPs could be a useful dietary supplement for raising the antioxidant status, growth parameters, haemato-immunological response, and whole-body composition of coho salmon fish.

## 1. Introduction

Considering fish contains high-quality protein and poly-unsaturated fatty acids (PUFA), it has a high nutritional value and is popular among consumers [1]. On the other hand, fish meat with a high PUFA content is susceptible to oxidation, which can result in a deterioration in quality. Certain plant polyphenol extracts have been utilized to improve the quality of flesh by preventing lipid oxidation [2]. In natural plant extracts, bioactive compounds such as polyphenols, alkaloids, and terpenoids are present in considerable quantities. These compounds have anti-inflammatory and antioxidant properties as their main biological roles. They are produced as secondary metabolites to protect cells against microbial infections [3]. A large and diverse class of chemicals is known as polyphenols. The two primary classes are represented by flavonoids and phenolic acids [4].

**Figure 1:**
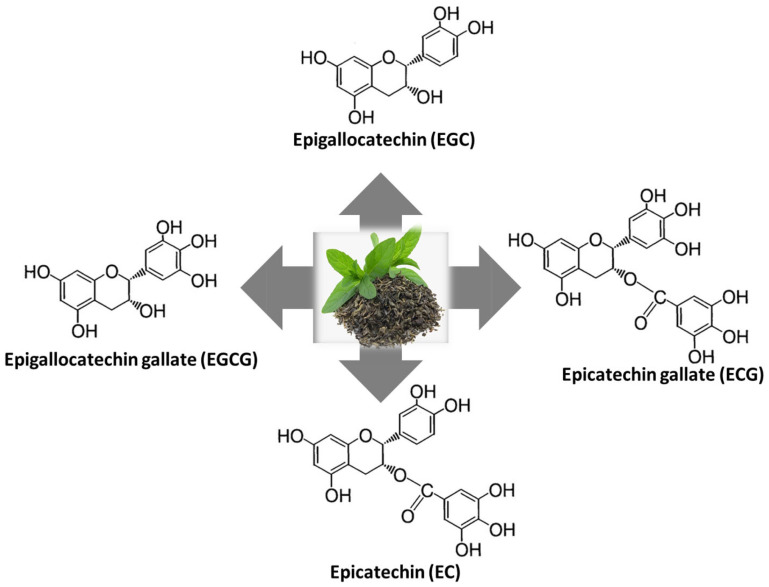
Major green tea polyphenols.

The health benefits of bioactive compounds found in green tea (*Camellia sinensis*) include anticancer, antioxidant, anti-inflammatory, anti-diabetes, anti-obesity properties, cardiovascular protection, immune regulation, and control of the intestinal microbiota [5,6]. Four primary tea catechins are found in tea leaves (Figure 1), which are known as epigallocatechin-gallate (EGCG), epigallo-catechin (EGC), epicatechin-gallate (ECG), and epicatechin (EC). Tea polyphenols (TPs) are a promising class of natural antioxidants [6]. ECG, EGC, and EC are the next highest catechins, with EGCG making up 59% of the total catechins [7]. According to Rothenberg et al. [8], TPs have been attributed to decreased visceral fat and enhanced weight loss in certain mammals and birds. Blood lipid parameters, such as reduced cholesterol and triglycerides, may also be impacted [9]. However, research on green tea’s bioactive compounds and their effects on cold-water fish is lacking. Potential benefits could include enhanced growth in tilapia (*Oreochromis niloticus*) [10], improved body composition in Asian seabass (*Lates calcarifer*) [11], lipid metabolism in zebra fish (*Danio rerio*) [12], immunity in Chinese rice field eel (*Monopterus albus*) [13], and rainbow trout (*Oncorhynchus mykiss*) [14]. 

Coho salmon (*O. kisutch*), one of the seven recognized Pacific salmons belonging to the *Oncorhynchus* genus, is widely distributed in the natural range and is important to both commercial and sport fisheries [15]. Approximately 120,000 metric tons of coho salmon are produced annually worldwide, mostly from aquaculture and the wild, with 80% of the cultured coho salmon being found in Chile and Norway [16]. In recent years, China has been cultivating coho salmon at an increasing rate. Unfortunately, research on coho salmon nutrition has only recently begun due to the growth of large-scale culture and an increase in feed demand. As a result, little is known about the dietary plant-based active substances that this species of fish requires. This study sets out to assess the impact of TPs on the growth, feed utilization, biochemical, haematological parameters, and immunity of coho salmon (*O. kisutch*). 

## 2. Materials and Methods

### 2.1. Feed Ingredients and Test Diets

Yu et al. [17] were followed in creating the basal diet as a control diet, which is displayed in Table S1. Table S2 lists the proximate compositions that are included in the diet. The supplier of TPs (>98% purity) was Jiangsu Caiwei Biotechnology Co., Ltd. in Suzhou, China. The other four experimental diets (within a 4.0-mm diameter) were created by supplementing the basal diet with 0%, 0.005%, 0.01%, 0.02%, and 0.04% TPs, which were based on earlier research [18].

### 2.2. Experimental Conditions

All experimental procedures were performed in accordance with the Weifang University Animal Care Advisory Committee. Coho salmon fish purchased from the Shandong Collaborative Innovation Centre of Coho Fish Health Culture Engineering Technology, Weifang, China, were used for these studies. The salmon had been farmed for two weeks in order for them to become acclimated to the test environment. For the current experiment, 300 coho salmon fish (180.51 ± 0.15 g) were selected, and they were randomized to five treatments (C, T1-T4). Three replicates of each treatment set of sixty fish (20 per tank) were stocked in tanks with a capacity of 240 L (0.8 × 0.6 × 0.6 m). Throughout the trial, the fish received nutrition twice a day, at 08:30 and 15:30 for ten weeks. The amount of feed given to the fish was around 4% of their body weight, and the leftover feed was collected about five minutes after the fish ceased eating. T1, T2, T3, and T4 gave TPs in place of 0.005%, 0.01%, 0.02%, and 0.04% of the basal diet, respectively, during ten weeks. Every two days, we made a 50% water change. The pH was roughly 7.0, the dissolved oxygen content was >7.0 mg/L, and the water temperature was maintained at between 13 and 15 °C throughout the experiment.

### 2.3. Growth Performance

The calculation formulae for the parameters mentioned above were as follows [19]:

Weight gain (WG, g) = Final weight − Initial weight

Relative growth rate (RGR, %) = 100 × (Final weight-initial weight)/initial weight

Specific growth rate (SGR) (% day 1) = 100 × (ln (mean final body weight) − ln (mean initial body weight))/days.

Daily growth rate (DGR, g/day) = (mean final weight − mean initial weight)/days

Feed efficiency (FE) (%) = 100 × (wet weight/dry feed intake).

Condition factor (CF) (%) = 100 × (wet weight/total length^3^)

Feed conversion ratio (FCR) = dry feed intake/wet weight gain 

Survival rate (%) = 100 × (final number of fishes/Initial number of fishes)

Hepato-somatic index (HSI, %) = 100 × (liver weight/body weight)

visceral-somatic index (VSI, %) = 100 × (visceral weight/body weight)

### 2.4. Sampling Procedures

Fish were fasted for 24 h before being counted and weighed in bulk in each replicate cage at the end of the 10-week feeding trial. Triclone methane sulfonate (MS-222) at a concentration of 20 mg/L was used to randomly sample and euthanize eight fish from each cage [20]. Three fish from each cage were sampled for morphological indices, such as condition factor (CF), visceral-somatic index (VSI), and hepato-somatic index (HSI). Following the measurement of body length, blood samples were obtained from the remaining five using a sterile, heparinized syringe in the caudal vein. The blood samples were then kept at room temperature for two hours. The serum from the samples was extracted and kept at −80 °C until the immunological and biochemical parameters were analyzed, following a centrifugation of 3500 g for 10 min at 4 °C. The three fish were quickly dissected after being bled, and the liver and muscles were taken out and preserved at −80 °C. Samples of muscle were utilized to analyze the muscle proximate composition, whereas samples of liver were used to determine antioxidative characteristics.

### 2.5. Proximate Composition Analysis

For the muscles and categorized diet groups, analyses of crude protein (Kjeldhal apparatus, nitrogen × 6.25), crude lipid (extraction with petroleum ether by Soxhlet apparatus), ash (incineration at 550 °C), and moisture (dried at 105 °C) were carried out [21]. 

### 2.6. Serum Biochemical Parameters

Diagnostic reagent kits (Jiancheng Biological Engineering Institute, Nanjing Engineering Institute, Nanjing, China) were employed to gauge the following biomarkers: glutamic-pyruvic transaminase (GPT), glutamic-oxalacetic transaminase (GOT), low-density lipoprotein cholesterol (LDL), high-density lipoprotein cholesterol (HDL), triglyceride (TG), total cholesterol (TC), alkaline phosphatase (ALP), and catalase (CAT). Operating instructions are followed in terms of reagent preparation, sample pretreatment, and operation technique [22]. 

### 2.7. Measurement of Antioxidant Status 

While the fish liver (*n* = 3) was dissected, it was homogenized for 30 to 45 s using a homogenizer in 100 mM phosphate solution (pH 7.4). The homogenizes were centrifuged for 30 min at 4 °C at 12,000× *g*. In accordance with Liu et al. [22], supernatants were gathered and kept at −80 °C. The kit instructions were followed to measure catalase (CAT), superoxide dismutase (SOD), and malondialdehyde (MDA) (Jiancheng Biological Engineering Institute, Nanjing Engineering Institute, Nanjing, China). 

### 2.8. Hematological Parameters

Using conventional heparinized micro-hematocrit capillary tubes, the hematocrit was determined via the micro-centrifuge technique. The coloring method of Natt and Herrick [23] was applied for RBC and WBC counts. After 30 min, a 1:50 ratio was used to mix the blood and color, and a hemocytometer was used to count the red and white blood cells [24]. The Sahli method was used to quantify the amounts of hemoglobin [24]. Erythrocyte metrics (MCV, MCH, and MCHC) were assessed using Imtiaz and Ahmad’s formula [24]. 

### 2.9. Immunological Parameters

The technique of Tukmechi et al. [25] was used to measure the lysozyme activity in serum. According to Villamil et al. [26], serum bactericidal activity against *Aeromonas hydrophila* (MTCC 1739) was determined. The level of respiratory burst content was measured according to Stasiack and Bauman [27]. 

### 2.10. Statistical Analysis

With SPSS 21.0 (SPSS Inc., Chicago, IL, USA), all data were examined. A one-way analysis of variance (ANOVA) and a Duncan multiple range test (DMRT) were used to compare the resultant data among the dietary groups. The data were deemed significant at *p* < 0.05, and the data were displayed as mean ± standard error (SE). 

## 3. Results

### 3.1. Growth, Feeding Performances, and Survival 

**Figure 2:**
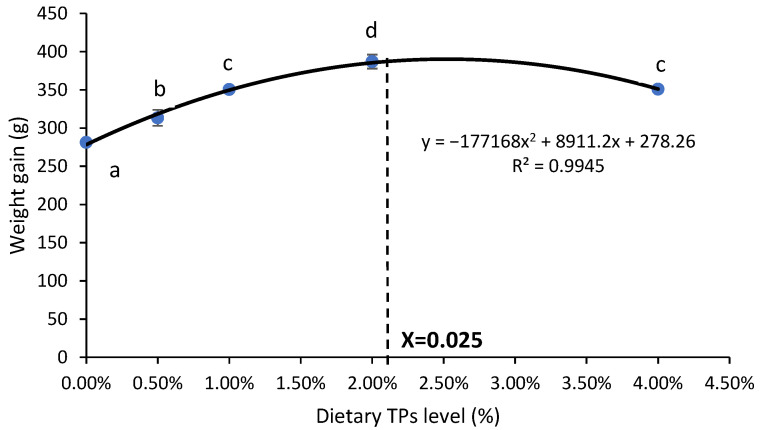

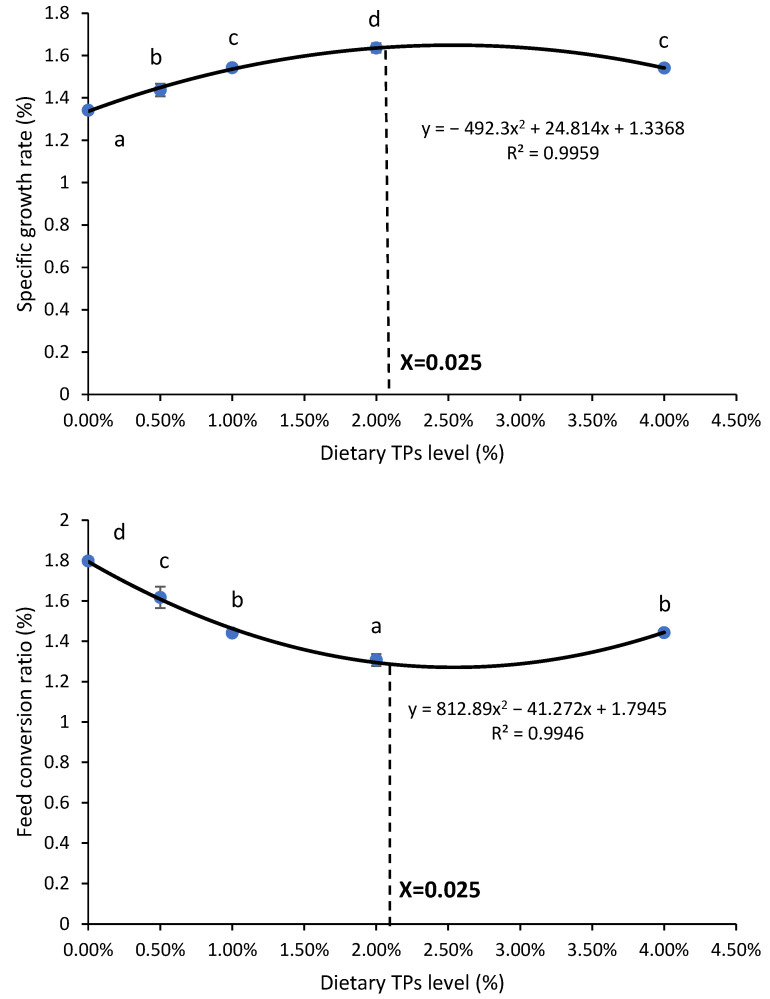
The polynomial regression analysis of WG, SGR, and FCR with dietary TP levels in coho salmon. The predicted dietary TP requirement of WG, SGR, and FCR is 0.025%. The data were expressed as means ± SE. Different letters (a–d) above the curves show significant differences among the treatments. SE, standard error of means.

As shown in Table 1, CF, HSI, VSI, and SR did not significantly differ between TP-fed and control fish (*p* > 0.05). When comparing the FW, FL, WG, RGR, DGR, SGR, and FE of the fish fed 0.02% of TPs, significant differences were found (*p* < 0.05). In the T2, T3, and T4 groups, there was no mortality noticed (*p* < 0.05). Weight gain (WG), specific growth rate (SGR), and feed conversion rate (FCR) were analyzed using polynomial regression while coho salmon were fed increasing amounts of dietary TPs for 10 weeks. WG, SGR, and FCR determined the dietary TPs needed to be 0.025% (Figure 2). 

### 3.2. Proximate Composition 

As Table 2 shows, the muscle moisture and ash content of coho salmon were not significantly affected by different dietary TP levels during the feeding trail (*p* > 0.05). The content of crude protein was lowest in the 0.01% TP-treated group (*p* < 0.05), compared with the control. The content of crude lipid was significantly increased by 0.02% (*p* < 0.05). 

### 3.3. Biochemical Blood Parameters

The findings indicated that the TP-fed and the control fish had significantly different GPT, LDL, TG, and CAT values (*p* < 0.05). GOT, HDL, TC, and ALP were not significantly changed between dietary regimens (*p* > 0.05) (Table 3). 

### 3.4. Antioxidant Enzyme Parameters

**Figure 3:**
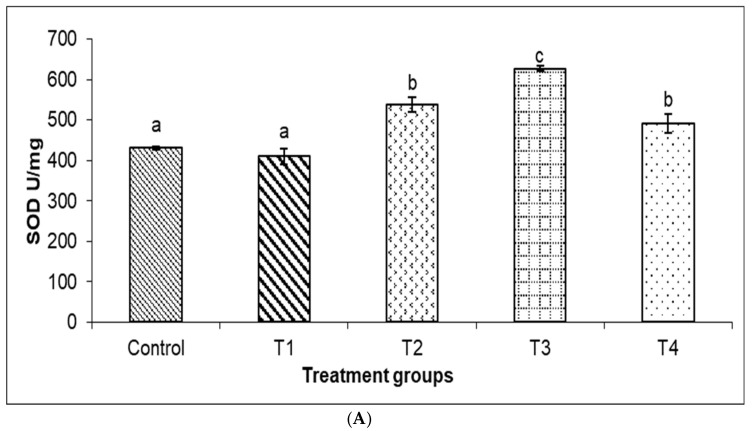

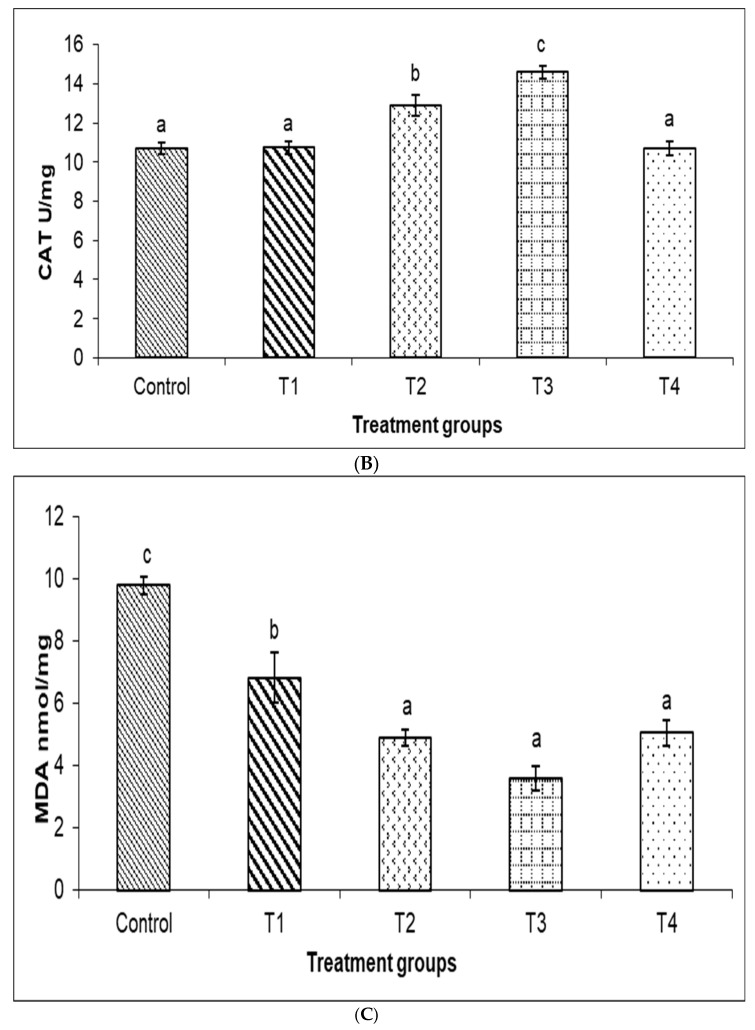
(**A**–**C**). The antioxidant enzyme (SOD: sodium dismutase; CAT: catalase; MDA: malondialdehyde) activity in the liver of coho salmon after 10 weeks of feeding with different levels of dietary TPs. Results are expressed as means ± SE (*n = 3*). Bars bearing different letters are significantly different by DMRT test (*p* < 0.05).

When comparing the TP-treated group to the control, the liver SOD and CAT activity dramatically improved (*p* < 0.05; Figure 3A,B). The MDA levels of TPs were substantially lower than those of the control fish (*p* < 0.05, Figure 3C).

### 3.5. Hematological Parameters

Table 4 shows the impact of TPs at several inclusion levels on the hematological parameters of coho salmon through food. According to the findings, there were no appreciable variations in MCH values between the TP-fed group and the control (*p* > 0.05). In comparison to the control, 0.02% of fish fed TP had significantly higher values of RBC, WBC, Hb, Hct, and MCV (*p* < 0.05). When comparing the control to the TP-treated fish, the MCHC levels were considerably greater (*p* < 0.05). 

### 3.6. Immunological Parameters

**Figure 4:**
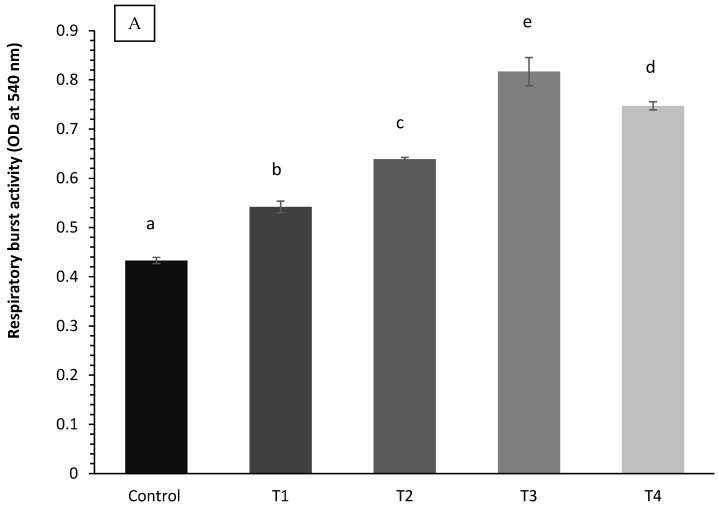

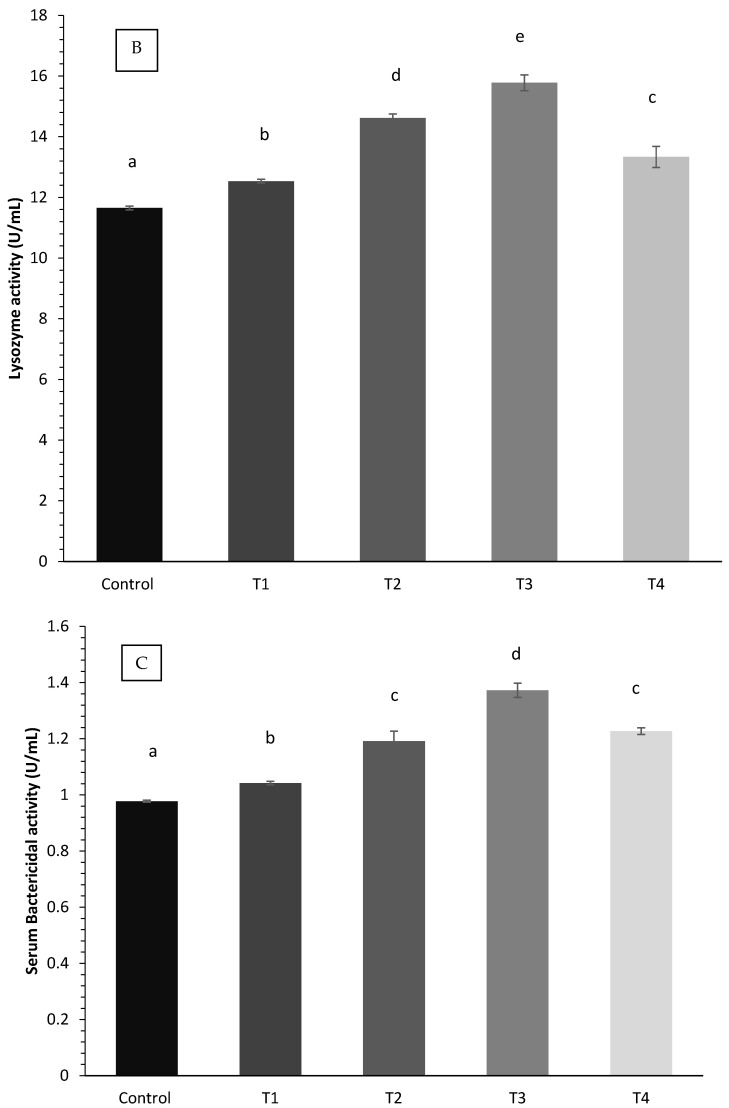
(**A**–**C**). The immunological response (A: respiratory burst activity; B: lysozyme activity; C: serum bactericidal activity) in coho salmon. Results are expressed as means ± SE (*n* = 3). Bars bearing different letters are significantly different by DMRT test (*p* < 0.05).

The serum immunological activity of coho salmon is shown in Figure 4A–C. In coho salmon fish, supplementing with TP greatly increased serum respiratory, lysozyme, and bactericidal activity (*p* < 0.05). In total, 0.02% of fish fed TP had significantly higher values of immunological parameters (*p* < 0.05).

## 4. Discussion

The beneficial ingredients found in tea residues, such as thiamine, tea saponins, and polyphenols, may improve animal growth performance [28]. After ten weeks of feeding, coho salmon fed with 0.02% of TPs demonstrated a significant improvement in weight measurements like WG, RGR, and DGR compared to control fish. On measurements of growth like SGR in coho salmon, dietary supplements had positive benefits. The findings demonstrate that TPs can raise SGR in fish fed 0.02% TPs from 1.34 ± 0.08 in the control group to 1.63 ± 0.02 in the treated group. The outcomes concur with the conclusions obtained by Welker et al. [14]. They [14] found that green tea had a growth-promoting effect on rainbow trout (*O. mykiss*) and that adding a lower dose of 0.1 to 0.5 g/kg of green tea to the diet allowed for the most effective growth and feed utilization. According to Yicong et al. [29], a dietary treatment of TP levels of 0.02% for 70 days did not significantly affect the growth performance of turbot (*Scopthalmus maximus*). Similarly, after four weeks of feeding, Ishihara et al. [30] found that adding 0.1% TPs to the meals of yellowtail (*Seriola quinqueradiata*) did not significantly change their WG. Comparable findings by Hwang et al. [31] revealed that green tea extract enhanced the growth performance of young black rockfish (*Sebastes schlegeli*), and they [31] drew the view that incorporating green tea extract into the diet may have improved the fish’s ability to absorb nutrients and thus improve nutrient utilization. Furthermore, large yellow croakers (*Larimichthys crocea*) exhibit enhanced growth performance and feed utilization when TPs are added to their diet at lower levels [32]. Conversely, just a few studies [29,30] found no evidence of any appreciable impact of TPs as a dietary supplement on cold-water fish growth performance.

In line with the findings presented here, TP supplementation at a dose of 50 mg/kg enhanced growth performance in tilapia (*O. niloticus*); however, TP treatment at a dose of 200 mg/kg reduced weight gain [33]. This implies that the quantity of TPs in fish diets has a dose-dependent influence on growth stimulation, with low TP dosages having a greater effect on accelerating fish growth performance and large TP dosages slowing down growth. To fully comprehend the mechanisms behind TPs’ effects on coho salmon growth, more investigation is needed.

Since the metabolism, transformation, and deposition of nutrients throughout an animal’s growth determine its body composition, the composition of its diet has a significant impact on the body composition of aquatic species. According to Lall and Tibbetts [34], a variety of factors, including the feeding system, diet components, and environmental conditions, affect the meat quality of fish. In this study, we evaluated fish quality using a variety of indicators, including moisture, ash content, crude protein, and crude lipid. The research reports that the tea extract supplement increased the protein and moisture content in freshwater angelfish (*Pterophyllum scalare)* [35] and had an impact on the black rockfish’s (*Sebastes schlegeli*) protein, moisture, fat, and ash [31]. Additionally, in juvenile black carp (*M. piceus*) fed TPs, the level of whole-body protein was significantly increased with respect to the control [18]. Juvenile olive flounder (*Paradichthys olivaceus*) [36] and grass carp (*Ctenopharyngodon idellus*) [37] fed green tea extracts showed no differences in body proteins and lipids between fed and control fish. In rainbow trout (*O. mykiss*) treated with tea EGCG, a similar tendency was seen [38]. Together, this finding suggests that various polyphenols may have distinct impacts on aquatic species based on the differences in their chemical composition. Although there are currently over 80,000 distinct polyphenols identified, it is estimated that only 10% of those that are actually found in nature have been thoroughly studied [39]. Our findings imply that dietary TPs have no detrimental effects on the body composition or flesh quality of coho salmon fish, as feeding TPs to the fish did not significantly alter their body composition.

The primary indicators used to assess liver injury are serum GOT and serum GPT. Damage to the liver results in increased absorbency of the cell membrane, transaminase enzyme secretion in the cells, and an increase in transaminase activity in the blood. Zhong et. al. [18] revealed that while GOT was considerably greater in the diet at 50 mg/kg, it was not significantly altered at low levels in black carp (*M. piceus)*. These findings suggest that TP overabundance may overwhelm the hepatic metabolism of coho salmon fish; until now, TP supplementation may have mitigated liver damage in giant yellow croaker (*L. crocea*) [32]. Compared to other fish, our data showed a decrease in the harm that lipid natural antioxidants caused to the liver, meaning that at low levels, the effect of TPs is not substantial. As an antinutritional factor, TPs might harm the liver if they are added in excess. Furthermore, in this experiment, TP supplementation had no effect on serum ALP compared to the control fish, indicating that TP supplementation may be able to partially ameliorate liver damage.

According to Ji et al. [32], TPs regulate TG, LDL, and HDL, which helps lower cholesterol and improve fat metabolism. TG is a kind of free lipid that is mostly prevalent in the body and acts as an indicator of how well the body is metabolizing fat. The two main lipoproteins with the highest cholesterol content are HDL and LDL. Whereas LDL transports cholesterol molecules from the liver throughout the body, HDL moves cholesterol from surrounding tissues into the liver, hence accelerating cholesterol metabolism. The cholesterol is conveyed and deposited in the artery wall when a glut of low-density lipoprotein oxidizes, further hardening the arteries. The TP supplement groups had considerably lower levels of TG, TC, LDL, and HDL, according to our data. While the trend of serum TG and LDL was similar to that of large yellow croakers (*L. crocea*), HDL was different [32]. This discrepancy may be because that marine fish has a higher lipid metabolism than freshwater fish [40]. Additionally, when TPs were supplemented, GPT and ALP were lower than in the control group, suggesting that TP supplementation may be able to prevent liver toxicity to some extent.

Liver antioxidant status is generally used to estimate whether the liver is diseased, injured, or functioning normally and is treated as a crucial component in maintaining liver barrier integrity to prevent chemical or pathogen infection [41]. TPs contain tri- and di-hydroxyl groups with strong antioxidant activity, which is deemed to be mainly related to their capacity to attenuate oxidative injury in organisms [42]. In this study, 0.01% and 0.02% TPs increased SOD and CAT activity in the liver. Furthermore, we use MDA, the primary product of lipid peroxidation, to track the status of lipid oxidation [43]. We showed that 0.02% TPs decreased MDA concentrations in the liver of coho salmon. Thus, our present results suggest that moderate TP doses can have indirect effects (by increasing enzyme activity) or direct effects (by increasing antioxidant activity) on antioxidant defense. In line with this finding, Nootash et al. [44] found that supplementation with green tea at 100 mg/kg increased SOD activity and reduced MDA concentration in the serum of rainbow trout (*O. mykiss*). Similarly, Guo et al. [45] found that selenium yeast with TPs produced the highest CAT activity in the serum of wuchang bream (*Megalobrama amblycephala*) and turbot (*Scophthalmus maximus*) [29]. In an 8-week feeding experiment aimed at investigating the effect of dietary TPs on antioxidants in juvenile black carp (*M. piceus*), MDA levels significantly decreased compared to the control [18]. In summary, the aforementioned results confirmed that supplementation with TPs might bring benefits to the antioxidant defense system of coho salmon. And it was speculated that dietary TPs could improve immunity and growth performance by enhancing liver antioxidant and barrier functions.

Haematological indicators are crucial instruments for assessing fish’s health [46]. Important elements of the non-specific cellular immune system, leukocytes can serve as a useful gauge of how fish react to their surroundings [47]. Leukocyte and hemoglobin levels have been shown to rise in reaction to stress in order to enhance the immune system and promote recovery [48]. According to the current study, there has been an increase in WBC levels in the blood of fish fed with TP-supplemented diets at the end of the study periods. Similarly, the higher WBC count was obtained in an Asian seabass (*L. calcarifer*) diet containing polyphenols at a dietary level of 0.5 g/kg compared with a fish-fed control diet [19]. In the present study, no significant variations in MCH were detected in TP-fed fish with respect to the control fish; however, in 0.02% TP treatment, blood hemoglobin was significantly lower than 0.005% and 0.01% TP treatment. Only RBC and WBC in the fish fed with 0.01 and 0.02% TPs were significantly higher than the control, while the hematocrit concentrations were significantly lower in 0.005% TP-fed and higher in 0.02% TP fed fish. Their action investigation appears to have had no effect on the hematological parameters in beluga juveniles (*Delphinapterus leucas*), except for an increase in leukocytes [49]. Leukocytes and blood hemoglobin concentrations were much lower in the cichlid *A. nigrofaciata* than in the control, although there were no discernible differences in RBC or hemoglobin content [50]. Finally, in sturgeon hybrid (*Huso huso* × *Acipenser ruthenus*) fed green tea extract, the number of leukocytes increased significantly in comparison to the control group [51]. 

Improving the immune response of fish maintained in protective circumstances is a helpful strategy to prevent diseases, infections, and consequently fish mortality [52]. Evidence has been accumulating in favor of an immunomodulatory effect of polyphenols [53,54,55]. While being absorbed by the gastrointestinal tract, polyphenols interact with the intestinal immune system, triggering the host to react defensively. Polyphenols can initiate signaling pathways that initiate an immunological response because they bind to receptors on a variety of cell types [56]. The study’s findings demonstrated that coho salmon treated with TPs had increased respiratory activity, lysozyme activity, and bactericidal activity in comparison to the control fish. Furthermore, optimal outcomes were obtained at 0.02% of the feed’s TPs, according to earlier research on rainbow trout (*O. mykiss*) [44,57], black rockfish (*Sebastes schlegeli*) [31], juvenile olive flounder (*Paralichthys olivaceus*) [36], tilapia (*O. niloticus*) [10], and kelp grouper (*Epinephelus bruneus*) [58].

## 5. Conclusions

In conclusion, our findings show that supplementing coho salmon (*O. kisutch*) diets with various levels of TPs enhances growth performance, feed efficiency, whole body proximate composition, and serum biochemical markers. Our results suggested that TPs could be a potential dietary supplement to improve the immunity and antioxidant level of coho salmon, and its optimal dosage was 0.02%, but it has negative effects on coho salmon if excessively supplemented. 

## Figures and Tables

**Table 1:** Growth and feeding performances of coho salmon fish fed TPs at different inclusion levels for 10 weeks.

Parameters	Control	T1	T2	T3	T4
IW (g)	180.43 ± 0.23	180.6 ± 0.20	180.33 ± 0.131	180.53 ± 0.17	180.7 ± 0.05
FW (g)	461.56 ± 2.23 ^a^	494.01 ± 10.6 ^b^	531 ± 4.06 ^c^	567.55 ± 9.14 ^d^	531.59 ± 3.17 ^c^
FL (cm)	32.33 ± 0.67 ^a^	35.33 ± 0.17 ^bc^	37.6 ± 0.55 ^d^	33.3 ± 0.75 ^ab^	36 ± 0.89 ^cd^
WG (g)	281.13 ± 2.46 ^a^	313.41 ± 10.52 ^b^	350.66 ± 3.94 ^c^	387.02 ± 9.10 ^d^	350.89 ± 3.21 ^c^
RGR (%)	155.81 ± 1.56 ^a^	173.53 ± 5.64 ^b^	194.45 ± 2.05 ^c^	214.37 ± 4.99 ^d^	194.18 ± 1.82 ^c^
DGR (g/day)	4.01 ± 0.03 ^a^	4.47 ± 0.15 ^b^	5.00 ± 0.05 ^c^	5.52 ± 0.13 ^d^	5.01 ± 0.04 ^c^
SGR (%/day)	1.34 ± 0.08 ^a^	1.43 ± 0.02 ^b^	1.54 ± 0.09 ^c^	1.63 ± 0.02 ^d^	1.54 ± 0.08 ^c^
FCR	1.79 ± 0.18 ^d^	1.61 ± 0.05 ^c^	1.44 ± 0.01 ^b^	1.30 ± 0.02 ^a^	1.44 ± 0.013 ^b^
FE (%)	55.65 ± 0.55 ^a^	61.97 ± 2.03 ^b^	69.44 ± 0.73 ^c^	76.56 ± 1.78 ^d^	69.35 ± 0.65 ^c^
CF (%)	1.37 ± 0.09	1.11 ± 0.018	1.00 ± 0.04	1.54 ± 0.12	1.14 ± 0.08
HSI (%)	1.21 ± 0.04	1.53 ± 0.088	1.47 ± 0.07	1.21 ± 0.22	1.43 ± 0.27
VSI (%)	7.12 ± 0.31 ^ab^	8.63 ± 0.58 ^b^	7.36 ± 0.50 ^ab^	5.84 ± 0.72 ^a^	7.24 ± 0.49 ^ab^
SR (%)	93.3 ± 6.67	96.6 ± 3.33	100 ± 0.00	100 ± 0.00	100 ± 0.00

Data were expressed as means ± SE. Different letters in each row show significant differences among dietary treatments by DMRT test (*p* < 0.05). SE, Standard Error of means (*n* = 3). IW: initial weight; FW: final weight; FL: final length; WG: weight gain; RGR: relative growth rate; DGR: daily growth rate; SGR: specific growth rate; FCR: feed conversion ratio; FE: feed efficiency; CF: condition factor; HSI: Hepato-somatic index; VSI: visceral-somatic index and SR: survival rate.

**Table 2:** Proximate analysis of the whole body of coho salmon fed the test diets with various levels of TPs for 10 weeks.

Parameters	Control	T1	T2	T3	T4
Crude lipid (%)	9.70 ± 0.007 ^b^	9.62 ± 0.03 ^ab^	9.23 ± 0.01 ^a^	9.74 ± 0.01 ^b^	9.67 ± 0.08 ^ab^
Crude protein (%)	14.15 ± 0.05 ^b^	14.21 ± 0.07 ^b^	13.61 ± 0.10 ^a^	14.28 ± 0.23 ^b^	13.93 ± 0.03 ^ab^
Ash (%)	2.71 ± 0.09	2.34 ± 0.04	2.35 ± 0.02	2.26 ± 0.05	2.30 ± 0.84
Moisture (%)	77.15 ± 0.09	77.00 ± 0.11	77.99 ± 0.16	76.94 ± 0.36	77.48 ± 0.06

Data were expressed as means ± SE. Different letters in each row show significant differences among dietary treatments by DMRT test (*p* < 0.05). SE, Standard Error of means (*n* = 3).

**Table 3:** Biochemical blood parameters of coho salmon fish fed TPs at different inclusions for 10 weeks.

Parameters	Control	T1	T2	T3	T4
GPT (U/mL)	6.82 ± 0.11 ^d^	5.02 ± 0.50 ^c^	1.89 ± 0.30 ^a^	0.90 ± 0.05 ^a^	3.04 ± 0.39 ^b^
GOT (U/mL)	15.30 ± 1.62	16.01 ± 1.60	14.78 ± 1.56	16.99 ± 1.00	11.89 ± 4.9
LDL (mg/dL)	1.60 ± 0.08 ^ab^	4.38 ± 0.40 ^d^	3.14 ± 0.56 ^c^	0.97 ± 0.10 ^a^	2.42 ± 0.122 ^bc^
HDL (mg/dL)	1.75 ± 0.16	1.43 ± 0.10	1.24 ± 0.11	1.03 ± 0.17	1.47 ± 0.40
TG (mg/dL)	1.76 ± 0.13 ^ab^	1.53 ± 0.09 ^abc^	1.21 ± 0.12 ^a^	1.39 ± 0.19 ^ab^	1.95 ± 0.15 ^c^
TC (mg/dL)	16.46 ± 1.78	12.57 ± 0.33	10.8 ± 1.20	10.06 ± 0.42	10.46 ± 4.10
ALP (U/mL)	3.41 ± 0.40	4.03 ± 0.25	3.67 ± 0.31	4.03 ± 0.24	3.28 ± 0.55
CAT (U/mL)	6.88 ± 0.40 ^a^	7.13 ± 1.39 ^a^	15.96 ± 0.66 ^b^	18.54 ± 0.16 ^b^	9.41 ± 3.62 ^a^

Data were expressed as means ± SE. Different letters in each row show significant differences among dietary treatments by DMRT test (*p* < 0.05). SE, Standard Error of means (*n* = 3). GPT: glutamic-pyruvic transaminase; GOT: glutamic-oxalacetic transaminase; LDL: low-density lipoprotein cholesterol; HDL: high-density lipoprotein cholesterol; TG: triglyceride, TC: total cholesterol; ALP: alkaline phosphatase, CAT: catalase.

**Table 4:** Hematological parameters of coho salmon fish fed TPs at difference inclusion levels for 10 weeks.

Treatment	Control	T1	T2	T3	T4
RBC (10^6^/mm^3^)	1.56 ± 0.60 ^b^	1.91 ± 0.01 ^ab^	2.14 ± 0.01 ^a^	2.24 ± 0.005 ^b^	2.11 ± 0.018 ^ab^
WBC (10^3^/mm^3^)	12.53 ± 0.11 ^b^	13.34 ± 0.09 ^b^	1.85 ± 0.04 ^a^	14.20 ± 0.03 ^b^	12.87 ± 0.24 ^ab^
Hb (g/dL)	8.86 ± 0.17 ^a^	9.27 ± 0.03 ^b^	10.33 ± 0.09 ^c^	11.19 ± 0.08 ^e^	10.75 ± 0.06 ^d^
Hct (%)	28.57 ± 0.03 ^b^	25.88 ± 0.32 ^a^	28.14 ± 0.40 ^b^	30.74 ± 0.40 ^c^	28.48 ± 0.02 ^b^
MCV (fl)	182.93 ± 7.17 ^b^	135.15 ± 1.17 ^a^	131.34 ± 1.02 ^a^	137.04 ± 1.79 ^a^	134.64 ± 1.13 ^a^
MCH (pg)	56.79 ± 3.10	48.45 ± 0.23	48.23 ± 0.38	49.74 ± 0.38	50.84 ± 0.69
MCHC (g/dL)	31.02 ± 0.64 ^a^	35.85 ± 0.45 ^a^	36.72 ± 0.37 ^b^	36.30 ± 0.21 ^a^	37.75 ± 0.26 ^ab^

Data were expressed as means ± SE. Different letters in each row show significant differences among dietary treatments by DMRT test (*p* < 0.05). SE, Standard Error of means (*n* = 3). RBC: red blood cells; WBC: white blood cells; Hb: haemoglobin; Hct: hematocrit; MCV: mean corpuscular volume; MCH: mean corpuscular haemoglobin; MCHC: mean corpuscular haemoglobin concentration.

## Data Availability

Data will be made available on request.

## Supplementary Materials

The following supporting information can be downloaded at https://www.mdpi.com/article/10.3390/ani14142104/s1: Table S1 shows the ingredients composition of basal diet and experimental diets. Table S2 shows the nutritional proximate composition of basal diet and experimental diets.
