# Supplementary material for: Impact of Nutritional Tea Polyphenols on Growth, Feed Efficiency, Biochemical Traits, Antioxidant Capacity, Haematological Parameters and Immunity in Coho Salmon (Oncorhynchus kisutch)
Source: Animals (Basel). 2024 Jul 18;14(14):2104. doi: 10.3390/ani14142104 (PMC11274171; doi:10.3390/ani14142104)
Supplement: Supplementary file 1 [file animals-14-02104-s001.zip › animals-3088577-supplementary.pdf]

animals

MDPI

Article

Impact of Nutritional Tea Polyphenols on Growth, Feed Efficiency, Biochemical Traits, Antioxidant Capacity, Haematological Parameters and Immunity in Coho Salmon (*Oncorhynchus kisutch*)

Hairui Yu [1,*,†], Govindharajan Sattanathan [1,†], Leyong Yu [1], Lingyao Li [2,3], and Yufang Xiao [1,*]

[1] Key Laboratory of Biochemistry and Molecular Biology, Weifang Key Laboratory of Coho Salmon Culturing Facility Engineering, Institute of Modern Facility Fisheries, College of Biology and Oceanography, Weifang University, Weifang 261061, China; sattanathanphd@gmail.com (G.S.); leyong618@gmail.com (L.Y.)

[2] Shandong Collaborative Innovation Center of Coho Salmon Health Culture Engineering Technology, Shandong Conqueren Marine Technology Co., Ltd., Weifang 261108, China; 980714742@163.com

[3] Conqueren Leading Fresh Science and Technology Inc., Ltd., Weifang 261205, China

* Correspondence: authors: yhr6003@hotmail.com (H.Y.); xiaoyf6@163.com (Y.X.)

† These authors contributed equally to this work.

Table S1 shows the ingredients composition of basal diet and experimental diets

Ingredients（%）	Treatment groups				
	Control	T1	T2	T3	T4
**Dry fish meal[1]**	55	55	55	55	55
**Shrimp meal powder[1]**	5	5	5	5	5
**Soybean flour[1]**	10	10	10	10	10
**Wheat Flour[1]**	18	18	18	18	18
**Cooked Starches[1]**	3.5	3.5	3.5	3.5	3.5
**Fish oil[1]**	3	3	3	3	3
**Soybean oil (v/w)[1]**	3	3	3	3	3
**Calcium dihydrogen phosphate[1]**	1	1	1	1	1
**Choline chloride VB4, 50%）[1]**	0.4	0.4	0.4	0.4	0.4
**Vc phosphate（35%）[1]**	0.1	0.1	0.1	0.1	0.1
**Cellulose[1]**	1.000	0.995	0.990	0.980	0.960
**Mineral mix（0.5%）[2]**	0.5	0.5	0.5	0.5	0.5
**Vitamin mix（0.25%）[3]**	0.5	0.5	0.5	0.5	0.5
**Tea polyphenols (%)**	0.000	0.005	0.01	0.02	0.04
**Total**	100	100	100	100	100

[1] Provided by Shandong Conqueren Marine Technology Co., Ltd., Weifang, China.

[2] Composition (g/kg mineral premix): $AlK(SO_4)_2{\cdot}12H_2O$, 123.7; $CuSO_4$.5H2O, 32.0; $CoCl_2{\cdot}6H_2O$, 49.0; $FeSO_4.7H_2O$, 707.0; $MgSO_4$.7H2O, 4317.0; $MnSO_4.4H_2O$, 31.0; KI, 5.3; NaCl, 4934.0; $Na_2SeO3.H_2O$, 3.4; $ZnSO_4.7H_2O$,177.0.

[3] Vitamin premix supplied the diets with (mg/kg dry diet): cholecalciferol, 0.04; α-tocopherol, 50; menadione, 44.0; thiamine-HCl, 12.0; riboffavin, 25.0; D-calcium pantothenate, 20.0; pyridoxine-HCl, 15.0; choline chloride, 500.0; meso-inositol, 200.0; D-biotin, 0.5; folic acid, 1.5; ascorbic acid, 100.0; niacin, 75.0; cyanocobalamin,0.01.

Table S2. shows the nutritional proximate composition of basal diet and experimental diets

**Chemical composition**		**Treatment groups**			
		**Control T1**	**T2**	**T3**	**T4**
**Crude Protein %**	44.90±0.001^{ab}	44.98±0.001^{ab}	45.40±0.003 ^{b}	44.09±0.002 ^{a}	44.43±0.004 ^{a}
**Crude Lipid %**	12.55±0.001^{c}	11.79±0.02 ^{a}	12.02±0.12 ^{ab}	12.22±0.13 ^{b}	12.10±0.001 ^{ab}
**Ash %**	12.097±0.10^{bc}	12.13±0.07 ^{d}	11.76±0.11 ^{a}	11.86±0.12 ^{ab}	11.70±0.008 ^{a}
**Moisture %**	11.23±0.39	11.26±0.37	11.25±0.51	11.23±0.26	11.04±0.26

Data were expressed as means±SE. Different letters in each row show significant differences among dietary treatments by DMRT (<0.05). SE, Standard Error of means (*n=3*).
